# Outbreaks of Tilapia Lake Virus Infection, Thailand, 2015–2016 

**DOI:** 10.3201/eid2306.161278

**Published:** 2017-06

**Authors:** Win Surachetpong, Taveesak Janetanakit, Nutthawan Nonthabenjawan, Puntanat Tattiyapong, Kwanrawee Sirikanchana, Alongkorn Amonsin

**Affiliations:** Kasetsart University, Bangkok, Thailand (W. Surachetpong, P. Tattiyapong);; Chulalongkorn University, Bangkok (T. Janetanakit, N. Nonthabenjawan, A. Amonsin);; Chulabhorn Research Institute, Bangkok (K. Sirikanchana);; Ministry of Education, Bangkok (K. Sirikanchana)

**Keywords:** Tilapia Lake virus, tilapia, Thailand, outbreaks, viruses, fish, aquaculture

## Abstract

During 2015–2016, several outbreaks of tilapia lake virus infection occurred among tilapia in Thailand. Phylogenetic analysis showed that the virus from Thailand grouped with a tilapia virus (family Orthomyxoviridae) from Israel. This emerging virus is a threat to tilapia aquaculture in Asia and worldwide.

Viral diseases are common causes of illness and death in cultured fish; such viruses include infectious salmon anemia virus, infectious hematopoietic necrosis virus, and viral hemorrhagic septicemia virus (*1*). With regard to tilapia, some viral pathogens, including betanodavirus, iridovirus, and herpes-like virus (*2*,*3*), reportedly cause severe disease. In recent years, Thailand has experienced extensive losses of tilapia; most losses occurred 1 month after transfer of fish from hatchery to grow-out cages in public rivers or reservoirs (1-month mortality syndrome). During routine investigation of this syndrome, multiple bacterial and parasitic infections were identified. However, no association was established between the outbreaks and any primary causative agent(s). Most deaths occurred within 2 weeks after the first dead fish were found. Similar observations of extensive losses of raised tilapia and wild fish in Israel and Ecuador have been reported (*4*,*5*). These outbreaks led to identification of a virus affecting tilapia, called tilapia lake virus (TiLV). The epidemiologic pattern and clinical signs for infected fish in Thailand led to suspicion of an illness of unknown etiology that was similar to TiLV infection. 

During 2015–2016, we investigated 32 outbreaks involving a large number of deaths of unknown cause among Nile tilapia (*Oreochromis niloticus*) and red hybrid tilapia (*Oreochromis* spp.). The outbreaks occurred at fish farms in central, western, eastern, and northeastern Thailand (Technical Appendix Figure 1). Affected fish were commonly found within 1 month after transfer from the hatchery facility to grow-out ponds or cages. In general, clinical signs and high mortality rates were associated with fish weighing 1–50 g (Technical Appendix Figure 2). Mortality rates among tilapia farms were 20%–90%; higher rates were associated with secondary bacterial and parasitic infections. Mortality rates peaked within 14 days after the first dead fish were found. 

As part of the outbreak investigation, samples of brain tissue were taken from fish at each of the 32 outbreak locations (each with a mortality rate >1%/day for 3 consecutive days): 10–30 moribund fish and 5–10 apparently healthy fish from the same culture areas. In total, 325 samples were collected and tested for etiologic agent(s) (*4*,*6*). Samples from fish involved in 22 of the 32 outbreaks were positive for TiLV (Technical Appendix Table 1). 

For our study, we selected a field sample positive for TiLV (designated TiLV/Tilapia/Thai/TV1/2016) and processed it for whole-genome sequencing. Another 6 TiLVs were selected for sequencing of the putative polymerase basic 1 (PB1) gene (Technical Appendix Table 2). TiLV genome sequencing was conducted by using newly designed primers based on reference TiLVs available in the GenBank database (*7*). Nucleotide sequences of 7 TiLVs from Thailand were submitted to GenBank (accession nos. KX631921–36). 

Comparison of the TiLVs from Thailand with those from Israel showed high nucleotide and amino acid identities (95.18%–99.10%). Among TiLVs from Thailand, nucleotide and amino acid identities for segment 1 or the putative PB1 gene of the virus were high (99.61%–100%) (Technical Appendix Table 3). Genetic analysis of the putative PB1 protein of TiLVs from Thailand and the viruses of the family *Orthomyxoviridae* showed that TiLVs from Thailand possessed motifs preA, A, B, C, D, and E similar to those of *Orthomyxoviridae* viruses, including influenza A, B, and C viruses; infectious salmon anemia virus; Dhori virus; and Thogoto virus (Technical Appendix Table 4) (*8*–*10*). Phylogenetic analysis showed that TiLVs from Thailand were closely related to TiLVs from Israel and grouped with the viruses of the family *Orthomyxoviridae* but not *Arenaviridae* and *Bunyaviridae* (Figure). This result suggests that the genetic composition of this emerging virus was similar to that of orthomyxoviruses and homologous with previously published TiLV sequences. 

**Figure:**
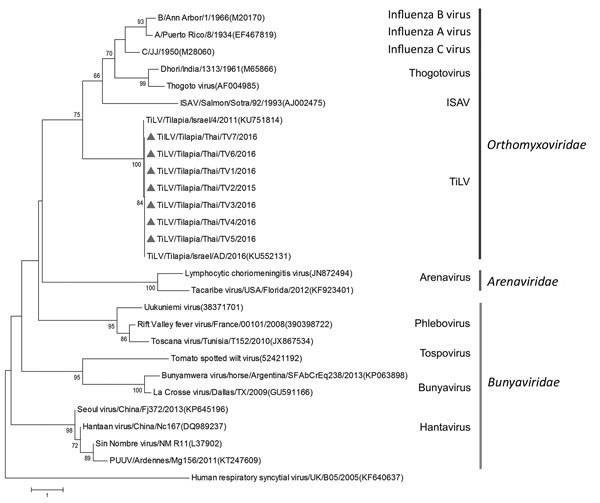
Phylogenetic analysis of the nucleotide sequences of RNA polymerase of TiLVs from Thailand (triangles) and reference viruses of the families Orthomyxoviridae, Arenaviridae, and Bunyaviridae. Genus and family groups are indicated; GenBank accession numbers are provided for reference viruses. The phylogenetic tree was constructed by using MEGA 6.0 (*10*) and applying a neighbor-joining bootstrap analysis (1,000 replications) with the Poisson model and gamma distribution. Human respiratory syncytial virus was used as an outgroup. ISAV, infectious salmon anemia virus; PUUV, Puumala virus; TiLV, tilapia lake virus. Scale bar indicates nucleotide substitutions per site.

Our PCR and whole-genome findings demonstrate genetic homology between TiLV from Thailand and the etiologic agent of a novel RNA virus infection of tilapia in Israel and Ecuador (*4*,*7*). Furthermore, the clinical signs and pathological presentation of infection with TiLV from Thailand are similar to those of infection with TiLV from Israel (Technical Appendix Figure 2). The clinical signs, gross lesions, and histopathologic lesions combined with virus identification and characterization highlight emerging TiLV in Thailand as the primary cause of the outbreaks. We also found that fish that survived massive die-offs rarely showed clinical signs, suggesting the development of specific immunity against the virus. It should be noted that the TiLVs from Thailand possessed 10 gene segments encoding 10 proteins, including segment 1 or putative PB1 protein. The pattern of protein motifs for this putative PB1 was similar to that for influenza viruses. To our knowledge, TiLV has infected tilapia only, no other aquatic or terrestrial animals. 

Our results emphasize that the virus isolated from Thailand shares high sequence similarity with TiLV from Israel, suggesting that this virus spreads across continents. Given that tilapia are the main aquaculture species, control of TiLV will be improved by further efforts such as strict biosecurity, vaccine development, and selection of resistant tilapia breeds. 

Technical Appendix Additional materials, methods, and results of study of tilapia lake virus outbreaks in Thailand, 2015–2016.
