# Supplementary material for: Outbreaks of Tilapia Lake Virus Infection, Thailand, 2015–2016
Source: Emerg Infect Dis. 2017 Jun;23(6):1031–3. doi: 10.3201/eid2306.161278 (PMC5443430; doi:10.3201/eid2306.161278)
Supplement: Technical Appendix — Additional materials, methods, and results of study of tilapia lake virus outbreaks in Thailand, 2015–2016. [file 16-1278-Techapp-s1.pdf]

Article DOI: https://dx.doi.org/10.3201/eid2306.161278

Outbreaks of Tilapia Lake Virus Infection, Thailand, 2015–2016

Technical Appendix

Materials and Methods

Clinical samples

Infected tilapia with clinical signs including loss of appetite, lethargy, swimming at the water surface, anemia, exophthalmia, abdominal swelling, and skin congestion and erosion were collected (Technical Appendix Figure 2). Internally, no specific gross pathological lesions were found. However, some fish developed brain congestion, pale gills and pale liver (Technical Appendix Figure 2). For external parasitic identification, the skin and gill samples were examined in wet mount under light microscope. For bacterial identification, anterior kidney samples were subjected to bacterial isolation using tryptic soy agar (TSA) or modified Shieh's agar. Bacterial species identification was performed using conventional biochemical test or API20NE test (BioMerieux, France). External parasitic identification findings were monogenean parasites (*Gyrodactylus* and *Dactylogyrus*) and ciliate protozoa (*Trichodina*). Bacterial findings were *Flavobacterium*, *Aeromonas* and *Streptococcus* (Technical Appendix Table 1).

Histopathology and Electron microscopy

For histopathological examination, brain, liver, spleen, heart, and kidney were collected from 3 fish (per outbreak) and kept in 10% buffered formalin. The samples were cut at 4 µm thick and processed for standard H&E staining. Histopathological findings were aggregation of lymphocytes and perivascular cuffing in brain tissue. For electron microscopic examination, the infected fish brains were filtered at 0.22 µm and prepared for EM transmission. Electron micrographs of negatively stained revealed enveloped virus particles with diameter between 50 to 80 nm (online Technical Appendix Figure 2).

Polymerase chain reaction

For molecular identification, PCR with specific primers for TiLV were performed (*1*). In addition, samples were tested for other viral infections including Betanodavirus and Iridovirus by specific PCR primers (*2*,*3*). In brief, RNA was extracted from brains (pooled sample of 3–5 brains) of infected and normal fish from the same culture area. RNA was subjected to PCR with TiLV specific primers.

Phylogenetic analysis

Phylogenetic analysis of TiLVs was conducted by comparing segment 1 (putative PB1 gene) of Thai TiLVs, Israel TiLV and reference viruses of Orthomyxoviridae, Arenaviridae and Bunyaviridae using program MEGA 6.0 (*4*).

References

1. Eyngor M, Zamostiano R, Kembou Tsofack JE, Berkowitz A, Bercovier H, Tinman S, et al. Identification of a novel RNA virus lethal to tilapia. J Clin Microbiol. 2014;52:4137–46. PubMed http://dx.doi.org/10.1128/JCM.00827-14

2. Bigarré L, Cabon J, Baud M, Heimann M, Body A, Lieffrig F, et al. Outbreak of betanodavirus infection in tilapia, Oreochromis niloticus (L.), in fresh water. J Fish Dis. 2009;32:667–73. PubMed http://dx.doi.org/10.1111/j.1365-2761.2009.01037.x

3. Kurita J, Nakajima K, Hirono I, Aoki I. Polymerase Chain Reaction (PCR) Amplification of DNA of Red Sea Bream Iridovirus (RSIV). Fish Pathol. 1998;33:17–23. http://dx.doi.org/10.3147/jsfp.33.17

4. Tamura K, Stecher G, Peterson D, Filipski A, Kumar S. MEGA6: Molecular Evolutionary Genetics Analysis version 6.0. Mol Biol Evol. 2013;30:2725–9. PubMed http://dx.doi.org/10.1093/molbev/mst197

5. Müller R, Poch O, Delarue M, Bishop DH, Bouloy M. Rift Valley fever virus L segment: correction of the sequence and possible functional role of newly identified regions conserved in RNA-dependent polymerases. J Gen Virol. 1994;75:1345–52. PubMed http://dx.doi.org/10.1099/0022-1317-75-6-1345

6. Poch O, Sauvaget I, Delarue M, Tordo N. Identification of four conserved motifs among the RNA-dependent polymerase encoding elements. EMBO J. 1989;8:3867–74. PubMed

7. Bacharach E, Mishra N, Briese T, Zody MC, Kembou Tsofack JE, Zamostiano R, et al. Characterization of a Novel Orthomyxo-like Virus Causing Mass Die-Offs of Tilapia. MBio. 2016;7:e00431–16. PubMed http://dx.doi.org/10.1128/mBio.00431-16

Technical Appendix Table 1. Description of TiLV outbreaks in Thailand*

				Laboratory diagnosis		
Outbreak	Date	Location	Species	Ectoparasite†	Bacteria identification‡	TiLV Identification§
1	15/10/2015	Ang Thong	RT	ND	ND	+
2	30/10/2015	Ang Thong	RT	ND	ND	+
3	11/11/2015	Ang Thong	RT	ND	ND	+
4	29/12/2015	Kanchanaburi	RT	ND	No growth	–
5	29/12/2015	Chai Nat	RT	ND	*Flavobacterium*	+
6	29/12/2015	Kanchanaburi	RT	ND	*Flavobacterium, Aeromonas*	+ (TV2)
7	29/12/2015	Chai Nat	RT	ND	*Flavobacterium*	–
8	05/01/2016	Nakhon Ratchasima	RT	1+	*Flavobacterium*	+ (TV3)
9	05/01/2016	Pathum Thani	RT	ND	No growth	+
10	15/01/2016	Pathum Thani	RT	2+	*Aeromonas*	+
11	15/01/2016	Chachoengsao	T	3+	*Aeromonas*	+ (TV4)
12	15/01/2016	Pathum Thani	RT	ND	ND	–
13	19/01/2016	Ratchaburi	RT	1+	*Aeromonas*	+ (TV5)
14	04/02/2016	Pathum Thani	RT	0	*Aeromonas*	+
15	05/02/2016	Kanchanaburi	RT	ND	*Aeromonas*	+
16	09/02/2016	Kanchanaburi	RT	1+	*Aeromonas*	+
17	16/02/2016	Samut Songkhram	RT	2+	ND	–
18	16/02/2016	Samut Songkhram	RT	3+	*Aeromonas*	+
19	18/02/2016	Pathum Thani	RT	3+	*Aeromonas*	–
20	26/02/2016	Pathum Thani	RT	2+	*Flavobacterium, Aeromonas*	+ (TV1)¶
21	27/02/2016	Samut Songkhram	RT	1+	No growth	+
22	30/03/2016	Pathum Thani	RT	ND	*Aeromonas*	+
23	28/04/2016	Nakhon Ratchasima	RT	ND	ND	+
24	28/04/2016	Pathum Thani	RT	ND	ND	+
25	06/05/2016	Pathum Thani	RT	2+	*Aeromonas*	+
26	06/05/2016	Prachin buri	T	0	*Streptococcus*	–
27	10/05/2016	Pathum Thani	T	1+	ND	–
28	13/05/2016	Nong Khai	T	3+	ND	–
29	20/05/2016	Phitsanulok	RT	0	*Aeromonas*	+ (TV6)
30	20/05/2016	Phitsanulok	T	0	*Streptococcus, Aeromonas*	–
31	23/05/2016	Chai Nat	RT	0	*Aeromonas*	–
32	24/05/2016	Khon Kaen	T	2+	*Aeromonas*	+ (TV7)

*__Outbreaks of massive tilapia death were investigated in 9 provinces during Oct 2015 to May 2016. Epidemiologic information and laboratory findings were shown__.
†Ectoparasite: External parasites were examined from skin and gills under light microscope. The majority of external parasites were monogenean parasites (*Gyrodactylus* and *Dactylogyrus*) and ciliate protozoa (*Trichodina*).
‡Bacterial Identification: Bacteria were isolated from anterior kidney and identified by conventional biochemical tests and API20NE test.
§TiLV Identification: Tilapia Lake Virus (TiLV) identification was performed by PCR with specific primers.
¶TV1 was subjected to whole genome sequencing.

Technical Appendix Table 2. List of Thai Tilapia lake viruses (TiLVs) characterized in this study*

Virus	Host species	Province	Date collection	Gene sequenced	GenBank accession no.
TiLV/Tilapia/Thai/TV1/2016	Red tilapia	Pathum Thani	Feb-2016	Whole genome Seg No. 1–10	KX631921 – KX631930
TiLV/Tilapia/Thai/TV2/2015	Red tilapia	Kanchanaburi	Dec-2015	Complete Seg No. 1	KX631931
TiLV/Tilapia/Thai/TV3/2016	Red tilapia	Nakhon Ratchasima	Jan-2016	Complete Seg No. 1	KX631932
TiLV/Tilapia/Thai/TV4/2016	Nile tilapia	Chachoengsao	Jan-2016	Complete Seg No. 1	KX631933
TiLV/Tilapia/Thai/TV5/2016	Red tilapia	Ratchaburi	Jan-2016	Complete Seg No. 1	KX631934
TiLV/Tilapia/Thai/TV6/2016	Red tilapia	Phitsanulok	May-2016	Complete Seg No. 1	KX631935
TiLV/Tilapia/Thai/TV7/2016	Nile tilapia	Khonkean	May-2016	Complete Seg No. 1	KX631936

*Seg No. 1: putative PB1.

Technical Appendix Table 3. Nucleotide and amino acid identities of Thai Tilapia Lake Virus (TiLV) against reference TiLV available in the GenBank database*

	Segment No., nucleotide (amino acid) identities, %									
Viruses	1<br>(1560 bp)	2<br>(1368 bp)	3<br>(1260 bp)	4<br>(1065 bp)	5<br>(1023 bp)	6<br>(954 bp)	7<br>(588 bp)	8<br>(525 bp)	9<br>(351 bp)	10<br>(342 bp)
Israel/4/2011	95.85<br>(99.22)	96.33<br>(98.73)	95.88<br>(99.52)	97.12<br>(99.15)	95.65<br>(97.90)	95.56<br>(96.74)	95.59<br>(97.37)	98.62<br>(99.42)	98.18<br>(97.35)	99.10<br>(99.11)
Israel/AD/2016	96.52<br>(99.42)	96.80<br>(99.49)	95.69<br>(99.27)	97.12<br>(99.43)	95.18<br>(97.90)	95.56<br>(97.74)	95.78<br>(96.83)	98.41<br>(100)	97.86<br>(96.43)	98.19<br>(98.20)
Thai/TV1/2016	100<br>(100)	100<br>(100)	100<br>(100)	100<br>(100)	100<br>(100)	100<br>(100)	100<br>(100)	100<br>(100)	100<br>(100)	100<br>(100)
Thai/TV2/2015	95.35<br>(99.81)	N/A	N/A	N/A	N/A	N/A	N/A	N/A	N/A	N/A
Thai/TV3/2016	95.42<br>(99.81)	N/A	N/A	N/A	N/A	N/A	N/A	N/A	N/A	N/A
Thai/TV4/2016	99.42<br>(100)	N/A	N/A	N/A	N/A	N/A	N/A	N/A	N/A	N/A
Thai/TV5/2016	95.70<br>(99.81)	N/A	N/A	N/A	N/A	N/A	N/A	N/A	N/A	N/A
Thai/TV6/2016	96.13<br>(99.61)	N/A	N/A	N/A	N/A	N/A	N/A	N/A	N/A	N/A
Thai/TV7/2016	95.84<br>(99.61)	N/A	N/A	N/A	N/A	N/A	N/A	N/A	N/A	N/A

*The number without blanket indicated % nucleotide identity and the number within blanket indicated % amino acid identity.
Note: Common amino acid variation of each segment. The alphabet before number indicates amino acid in Israel TiLV. The number indicates a position of amino acid variation. The alphabet post number indicates amino acid in Thai TiLV. Putative PB1: K446R, Segment No.2: K6R and A231T, Segment No.3: V275M, Segment No.4: S24G and A33V, Segment No.5: I17M, D109E, R155K, T163A, V291I and L294F, Segment No.6: L13F, K55I, M95R, R159H, K274R, N276D and I301M, Segment No.7: S22I, E72R, H113R, C177S and K189E, Segment No.8: V45I, S66G and R114K, Segment No.9: V45I,S66G and R114K, Segment No.10: R89I.

Technical Appendix Table 4. Summary of Motifs preA, A, B, C, D and E of Orthomyxoviridae PB 1 aa alignment (*5, 6*)*

Viruses	Premotif A	Motif A	Motif B	Motif C	Motif D	Motif E
IAV	KDAERGKLKRRAIATPGM<br>QIRGFVYFVET	TELSFTITGDNT<br>KWNENQN	ASLSPGMMMGMF<br>NMLSTVLGVS	TYWWDGLQ<br>SSDDFAL	GINMSKKK<br>S-YINR	TGTFEFTSF<br>FYR
IBV	KDAERGKLKRRAIATAGIQI<br>RGFVLVVEN	GGISMTVTGDN<br>TKWNECLN	ASLSPGMMMGMF<br>NMLSTVLGVA	EYLWDGLQ<br>SSDDFAL	GINMSKKK<br>S-YCNE	TGMFEFTSM<br>FYR
ICV	KDGERGKLQRRAIATPGMI<br>VRPFSKIVET	DQFAVNITGDN<br>SKWNECQQ	CFLPGGMLMGMF<br>NMLSTVLGVS	GCFWTGLQ<br>SSDDFVL	GINMSLEKS<br>-YGSL	PELFEFTSM<br>FFD
ISAV	KNSERTKLEPRAVFTAGVP<br>WRAFIFVLEQ	GQTLVTLTGDN<br>SKYNESMC	IRVRRGMLMGMA<br>NNAFTTASTI	PEAVYTLQS<br>SDDFVT	GLNVSQKK<br>SFYVEG	TT-<br>FEFNSMFVR
Dho	KHLERGRLNRRTIATPSML<br>ARGFVKIVED	SEVTGELSGDQ<br>EKFNECLD	IRCTLGMFMGMFN<br>LSSTLLALI	EITGDHVES<br>SDDFIH	GINMSPSK<br>CILISP	AGIGEFNSK<br>YHH
Tho	KHLERGRLNRRTIATPSML<br>IRGFVKIVED	TAVTGELSGDQ<br>EKFNECLD	ISCRLGMFMGMYN<br>LTSTLLALI	ELTGSHVES<br>SDDFIH	GINMSPSK<br>CILISP	AGIGEFNSK<br>FHH
TiLV4–11	RDQERGKPKSRAIFLSHPF<br>FRLLSSVVET	ESRKHVLNGDC<br>TKYNEAID	—-<br>GGMLMGMFNATA<br>TLA—	GTTDRFLSF<br>SDDFIT	–NLSLKKS-<br>YISV	AS-<br>LEINSCTLT
TiLVAD-16	RDQERGKPKSRAIFLSHPF<br>FRLLSSVVET	ESRKHVLNGDC<br>TKYNEAID	—-<br>GGMLMGMFNATA<br>TLA—	GTTDRFLSF<br>SDDFIT	–NLSLKKS-<br>YISV	AS-<br>LEINSCTLT
Thai TiLV	RDQERGKPKSRAIFLSHPF<br>FRLLSSVVET	ESRKHVLNGDC<br>TKYNEAID	—-<br>GGMLMGMFNATA<br>TLA—	GTTDRFLSF<br>SDDFIT	–NLSLKKS-<br>YISV	AS-<br>LEINSCTLT

*Gap represented by -. Residues that are invariant for all RNA polymerases are shown in Bold. Conserved residues among negative-stranded RNA are shown in bold and underline. Description of viruses are as follows: IAV: influenza A/Puerto Rico/8/1934 (EF467819), IBV: influenza B/Ann Arbor/1/1966 (M20170), ICV: influenza C/JJ/1950(M28060), ISAV: Infectious salmon anemia virus strain Sotra 92/93(AJ002475), Dho: Dhori virus (M65866), Tho: Thogoto virus (AF004985).

Technical Appendix Table 5. List of Oligonucleotide primers for Thai TiLV sequencing*

Segment	Primer Name	Primer Sequence (5′ – 3′)	Bp	Product size (Bp)
1	TiLV1F	CCAAACGTTATCTCTTAATTACGCAC	26	1641
	TiLV1R	GCAAATATTTCTCTCATTCGCCT	23	
2	TiLV2F	ACTCTCTATTACCAAATACATTTACT	26	1445
	TiLV2R	TTACCATATATATAGTGAAGGC	22	
3	TiLV3F	ACCCCTTAATCCTTAATAGACCGTTA	26	1352
	TiLV3R	CCCATAATCCTCTATTAGAACGTCGT	26	
4	TiLV4F	CCAAAGTTTACTCCTATTACCCAGA	25	1250
	TiLV4R	GCAAATCTTTCTCCAATTACCGTCT	25	
5	TiLV5F	CCAAATGTTTCTCTTATCTCAGACTC	26	1087
	TiLV5R	CTTTTTCTCAGTTTACCACTTTATG	25	
6	TiLV6F	CCAAATTTTACCTCTCGCAT	20	1027
	TiLV6R	TCAAGCACTTAAAACTGTACC	21	
7	TiLV7F	CTCTCTTTGCATTGCATACCGT	22	704
	TiLV7R	GACCAATTATCCCTGCTTTCA	21	
8	TiLV8F	ACCTCATCTACACTAACATTTCCA	24	637
	TiLV8R	TCATCATTACACAAATGGAGTAGCT	25	
9	TiLV9F	ACAAGTCCGATTACTTTTTCCGC	23	530
	TiLV9R	TCTTTCTCACGTCCTTAAAGTCA	23	
10	TiLV10F	AACCCTACTAACACCAAATATAGCT	25	450
	TiLV10R	CTTTCCCTCTGACACCCTGT	20	

*Primers were designed based on available TiLV reference (7).

Technical Appendix Table 6. Detail description of genome composition of Thai TiLV (TV1)

	Israel TiLV, TIL4/2011*		Thai TiLV, THA1/2015	
Contiq	Segment length (Bp)	Predicted protein (AA)	Segment length (Bp)	Predicted protein (AA)
1	1,641	519	1562 (1560)	519
2	1,471	457	1368 (1368)	455
3	1,371	419	1301 (1260)	419
4	1,250	356	1170 (1065)	354
5	1,099	343	1024 (1023)	340
6	1,044	317	988 (954)	317
7	777	195	685 (588)	195
8	657	174	588 (525)	174
9	548	118	484 (351)	116
10	465	113	405 (342)	113

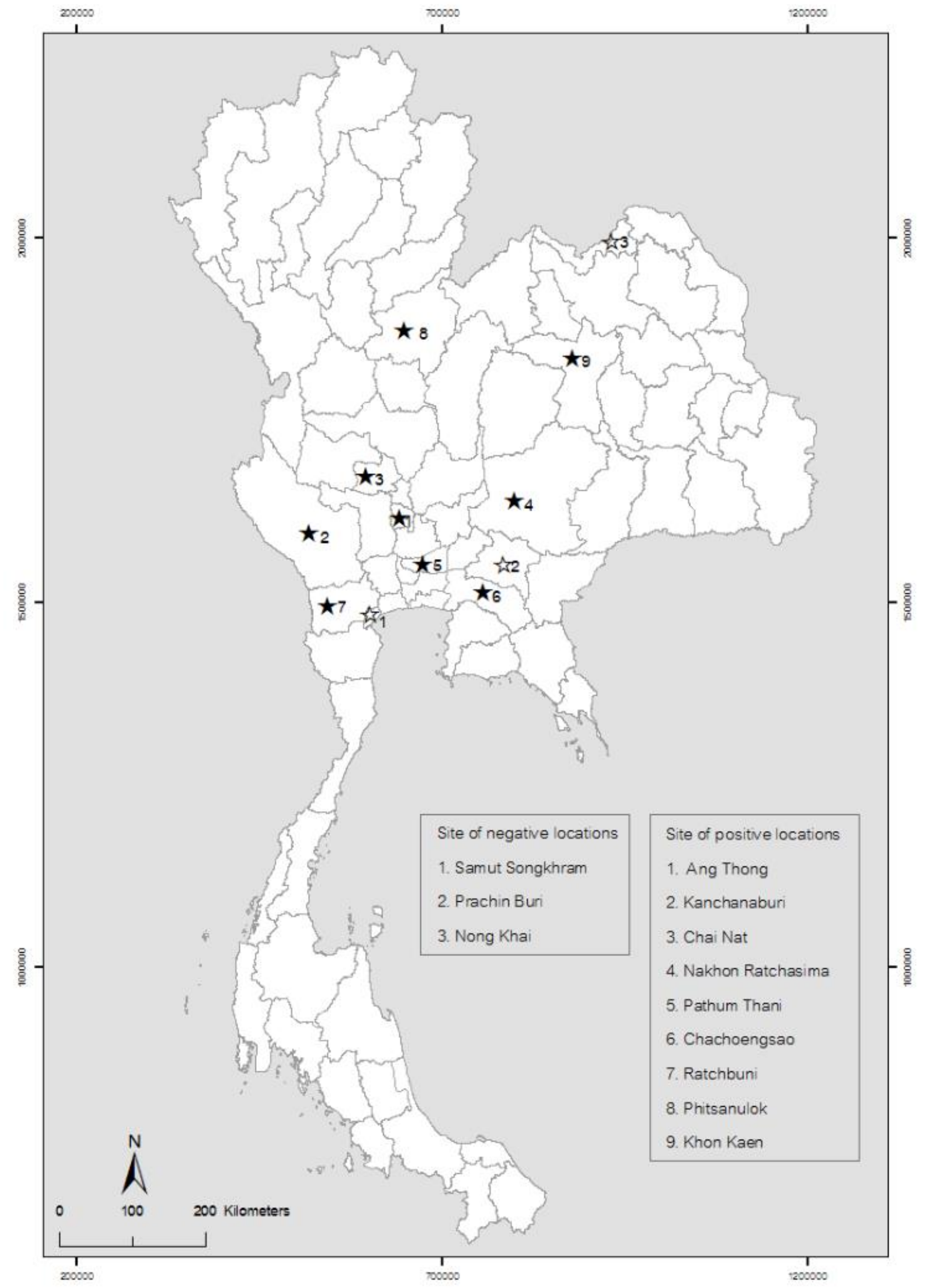

Technical Appendix Figure 1. Locations of sample collection covering central, eastern, northeastern and western of Thailand. Stars (solid) represent site of virus positive location.

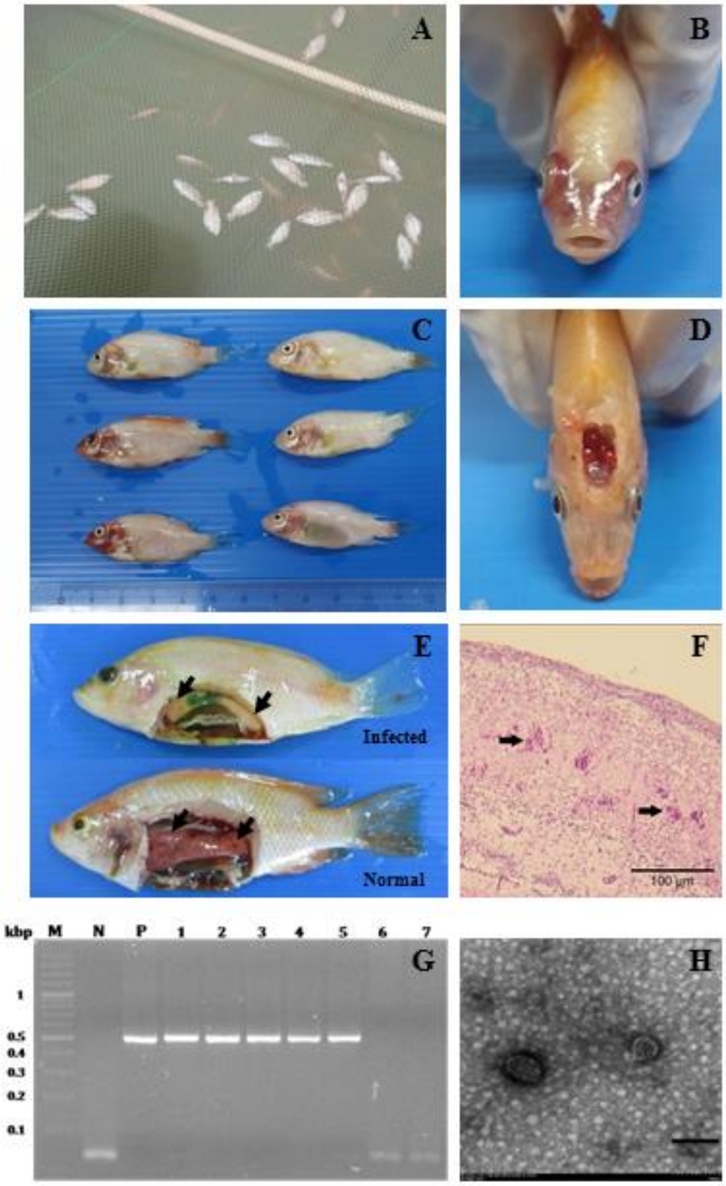

Technical Appendix Figure 2. Gross and histopathological lesions of infected tilapia, Thailand. (A) Massive losses of fish due to mortality at 2 weeks post transfer into cages. (B and C) Gross appearance of infected tilapia included skin congestion and mild exophthalmia, (D and E) brain congestion and pale liver. (F) Histological findings showed influx of mononuclear lymphocytes in the brain consistent with non suppurative meningoencephalitis with multifocal hemorrhage. (G) PCR identification of TiLV from infected fish. M = marker, N = negative, p = positive control (plasmid containing TiLV fragment), Lane 1–5: brain from moribund fish, Lane 6–7: brain from normal fish. Samples were pools of 3 brains. (H) Morphology of virus prepared from infected brain. The virion size is 50–80 nm with electron dense aggregate surface; bar size = 50 nm.
